# Perspectives on gastroesophageal reflux disease in primary care: the REFLEX study of patient-physician agreement

**DOI:** 10.1186/1471-230X-11-25

**Published:** 2011-03-24

**Authors:** Etienne Dorval, Jean-Francois Rey, Christine Soufflet, Katarina Halling, Philippe Barthélemy

**Affiliations:** 1 Gastroenterology, Trousseau Hospital, Tours, France; 2 Hepatology and Gastroenterology, Institut Arnault Tzanck, Saint Laurent du Var, France; 3 Medical Department, AstraZeneca, Rueil Malmaison, France; 4 AstraZeneca R&D Mölndal, Sweden; 5 PRO Consulting Europe, Gothenburg, Sweden

## Abstract

**Background:**

Physicians may be unaware of the severity and extent of gastroesophageal reflux disease (GERD) in their patients. The aim of this study was to evaluate patient-physician agreement concerning proton pump inhibitor (PPI) treatment.

**Methods:**

1818 French primary-care physicians and 5174 adult patients with GERD who were taking PPIs answered questions regarding symptoms and treatment satisfaction. Patient-physician agreement was scored using the Kappa (κ) method.

**Results:**

There was moderate patient-physician agreement for PPI treatment satisfaction (κ = 0.60), PPI prescription adherence (κ = 0.57) and use of over-the-counter gastrointestinal medications (κ = 0.44-0.51). Patient satisfaction with PPI therapy and PPI treatment adherence rates were both ~90%. There was poor patient-physician agreement concerning PPI therapy expectations (κ = 0.22-0.33). Residual reflux symptoms occurred in 61% of patients. Physicians underestimated residual symptom severity compared with their patients (κ = 0.43-0.47), though there was good agreement regarding the presence (κ = 0.62-0.78) and frequency (κ = 0.61-0.66) of these symptoms and their effect on patients' daily life (κ = 0.64).

**Conclusions:**

Patient-physician agreement regarding PPI therapy for GERD was moderate or good for the presence of residual symptoms and moderate for treatment satisfaction, but poor for treatment expectations. PPI treatment resulted in high satisfaction rates, but residual symptoms were fairly common and their severity was underestimated by physicians.

## Background

Gastroesophageal reflux disease (GERD) develops when reflux of the stomach contents into the esophagus causes troublesome symptoms such as heartburn or acid regurgitation, and/or complications such as esophagitis [1]. The prevalence of GERD is thought to be 10-20% in Western countries based on the presence of heartburn and/or regurgitation at least once per week in general population surveys [2]. In France, 8% of the population experience typical symptoms of GERD at least once per week [3].

GERD is a chronic disease and disrupts many aspects of patients' everyday lives. At least two-thirds of patients still have GERD 10 years after their initial diagnosis, and almost half of adults with GERD have had their symptoms for 10 years or more [2,4]. Surveys of patients and the general population using validated generic health questionnaires show that GERD disrupts patients' lives in many ways, primarily by causing pain [5,6], but also through interference with normal activities such as eating and drinking, work, sleep and enjoyment of social occasions [7].

Consultation rates for GERD are low: only 5-30% of individuals with gastroesophageal reflux consult a physician about their symptoms each year [8-10]. Symptom burden and anxiety about serious underlying disease are the major reasons for consultation [3,11,12]. Only 1.7% of primary-care consultations are the result of GERD [13], but consultation rates are increasing, probably because of an apparently increasing prevalence of GERD in the community [13,14].

Physicians are not always aware of the full burden of illness of patients who consult them with reflux symptoms. A study of patient and physician ratings of reflux symptoms in clinical trials showed that physicians tend to underestimate the prevalence and severity of such symptoms [15]. Agreement between patients and physicians was better after treatment than before, possibly because physicians' ratings were more likely to agree with those of patients when symptoms were absent [15]. A study in primary care has shown that patients do not provide their physicians with full information on their reflux symptoms unless they have the aid of a questionnaire [16], and that physicians find such information very useful [17].

Recent surveys in primary care indicate that approximately a quarter of patients are not satisfied with their prescription treatment for GERD [18,19]. Patients with GERD continue to experience residual reflux symptoms despite acid-suppressive therapy, and these symptoms can substantially reduce patients' quality of life [11]. This leads to use of over-the-counter (OTC) medications such as antacids, alginates and histamine (H_2_)-receptor antagonists, repeated physician consultation and treatment dissatisfaction [3,11,20,21]. Unsurprisingly, patients with the greatest burden of illness during treatment are least likely to be satisfied with their treatment [22].

The aim of the present study was to evaluate patient-physician agreement concerning treatment satisfaction in a large population of adult patients with GERD treated with proton pump inhibitors (PPIs) in primary care. This study also assessed the presence, frequency and severity of reflux symptoms, as well as their effect on patients' daily life and the need for self-medication with OTC gastrointestinal drugs.

## Methods

The study was conducted from 1 September 2003 to 22 June 2004. A representative sample of 2500 primary-care physicians working in metropolitan France was selected by drawing lots from an independent database with regional stratification. The following information was collected about the physicians: sex, age, average number of adult GERD patients, number of patients seen each week and GERD treatment patterns. The patients who were asked to participate were the first adult patients coming to consult their physician (for any reason) after enrolment of the centre in the study, who had reflux symptoms, a PPI prescription covering the previous month and who had taken their PPI on at least 14 days of that month.

Patients were excluded from the study if they were under 18 years old, were unable to fill in the questionnaire, did not have reflux symptoms or esophagitis, were not treated in the last month with a PPI prescribed in a continuous way (or had not taken a PPI for at least 14 days in the preceding month), or were enrolled before the designated inclusion period.

The patient questionnaire collected information including demographic data (age, sex, weight, height, and alcohol and tobacco consumption); recent and current PPI treatment (agent, dosage, start date and mode [continuous, intermittent or on-demand]); upper gastrointestinal endoscopy in the previous 3 years (presence or absence of esophagitis); specialist consultations about GERD in the previous 3 years; concurrent treatment with non-steroidal anti-inflammatory drugs (NSAIDs), aspirin, anticoagulants and corticosteroids.

### Assessment of symptoms and satisfaction

The patient and the physician each answered questions about the symptoms and treatment satisfaction. In the case of the patient questionnaire, questions were asked regarding patients' own experiences and satisfaction. With regard to the physician questionnaire, doctors were asked about their degree of satisfaction with the PPI treatment of their patient, and their own knowledge of their patients' quality of life and residual symptoms. The patient and physician questionnaires both asked for details on the following items (with the symptom descriptors used in the patient questionnaire given in brackets):

• the presence/absence (at any time), frequency (number of days per week the symptoms were experienced) and severity (mild, moderate or severe) of daytime heartburn (a burning feeling rising from the stomach or lower chest up towards the neck), night-time heartburn and acid regurgitation (a sour or bitter liquid in the mouth) during treatment with PPIs

• the presence/absence of other gastrointestinal symptoms (at any time), including dysphagia (difficulty swallowing), epigastric burning (burning in the stomach), abdominal pain (pain in the stomach), nausea (desire to vomit), vomiting, eructation (belching) and bloating (distension) during treatment with PPIs

• current expectations with respect to treatment, the current impact of reflux symptoms on everyday life and current overall patient satisfaction

• adherence to PPI therapy (yes/no)

• self-medication for reflux symptoms during treatment with PPIs (agents and frequency).

Patients received an information form concerning the study and were free to choose not to participate. Formal written informed consent was not required because the investigation did not affect normal medical care or modify the doctor-patient relationship. Each doctor saw their patients within the usual framework of consultations, which were not convened specifically for the purposes of the investigation. Each patient completed his or her questionnaire anonymously and sealed it in an envelope, which along with the physician-completed questionnaire, was sent for analysis to Axonal S.A. (Nanterre, France), a contract research organization responsible for the study logistics. Additional assessment and explorations were not required by this protocol. The investigation was the subject of a declaration specific to the Advisory Committee for Data Processing in Health Research (Comité consultatif sur le traitement de l'information en matière de recherche dans le domaine de la santé) and the approval of the National Commission of Data Processing and Freedoms (Commission Nationale de l'Informatique et des Libertés). The study also complied with epidemiological good practice according to the recommendations of the Association of French-Speaking Epidemiologists (ADELF). The study was approved by the National Physician Council in July 2003. Ethical approval was not required.

### Statistics

The presence, frequency and severity of reflux symptoms were defined on the basis of patient rather than physician reports [23]. The effect of GERD on everyday life, overall satisfaction and expectations with respect to treatment (symptoms, quality of life and self-medication) was assessed through a five-grade Likert scale. Physician-patient answers were analysed by the Kappa (κ) method [24], using the following grades: very poor (0.00-0.20), poor (0.21-0.40), moderate (0.41-0.60), good (0.61-0.80) and excellent (0.81-1.00).

## Results

### Study population

In total, 1818 primary-care physicians participated in the study, giving a response rate of 72.7%. The physicians were predominantly male (86.2%), and their mean age (± SD) was 47.0 ± 7.1 years. They saw 122.1 ± 50.4 adult patients each week, of whom 13.1 ± 9.4 were consulting them about gastroesophageal reflux symptoms. With respect to prescribing practice, most physicians prescribed PPIs often (68.5%) or always (29.1%) for adult patients with GERD, whereas H_2_-receptor antagonists were prescribed rarely (46.8%) or never (17.2%).

Overall, 1818 physicians recruited 5326 patients to the study. The demographic and clinical characteristics of the patients recruited to the study are summarized in Table 1. Paired patient-physician data were evaluable in 5174 cases and were included in the study analyses.

**Table 1:** Patient sociodemographic and clinical characteristics (*N *= 5326).

Characteristic	Mean ± SD or %
Age (years, mean ± SD)	53.0 ± 14.6
Sex (% male)	57.4
Weight (kg, mean ± SD)	74.8 ± 13.6
Height (cm, mean ± SD)	169.4 ± 8.4
BMI (kg/m^2^, mean ± SD)	26.0 ± 4.07
Smokers* (%)	37.0
Alcohol consumers* (%)	52.2
PPI use	
Continuous (%)	68.8
Intermittent (%)	20.1
On-demand (%)	11.0
Use (often or always) of OTC medication according to the physician	
Antacids and alginates (%)	8.2
Prokinetics (%)	3.4
H_2_-receptor antagonists (%)	0.8
Upper GI endoscopy in previous 3 years (%)	44.9
Esophagitis detected on endoscopy (%)	77.3
Concurrent medication	
NSAIDs (%)	13.1
Aspirin (%)	7.7
Anticoagulants (%)	6.8
Corticosteroids (%)	1.6

*Includes occasional and regular use.

BMI, body mass index; GI, gastrointestinal; H_2, _histamine; NSAID, non-steroidal anti-inflammatory drug; OTC, over-the-counter; PPI, proton pump inhibitor; SD, standard deviation.

### Patient expectation, adherence and satisfaction

**Figure 1:**
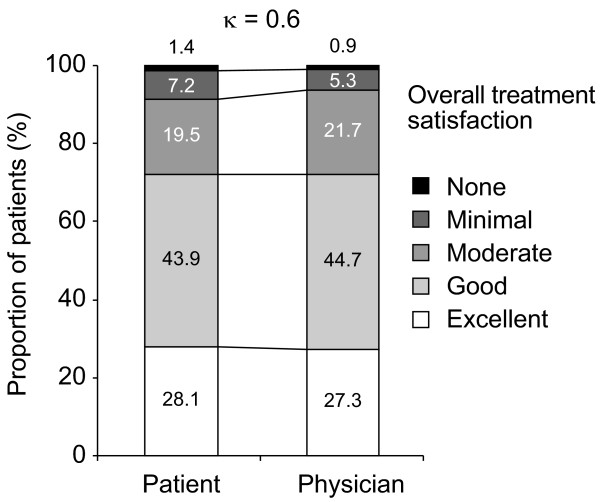
**Patient and physician ratings of patient satisfaction with treatment**.

Most patients (72.0%) rated their satisfaction with their PPI treatment for GERD to be excellent (28.1%) or good (43.9%). A further 19.5% reported moderate satisfaction, 7.2% minimal satisfaction and 1.4% no satisfaction (Figure 1). Similarly, physicians rated patient satisfaction with PPI treatment as excellent for 27.3% of their patients and good for 44.7%. This translated into moderate agreement between patients and physicians with respect to overall treatment satisfaction. In subgroup analyses, this agreement was not influenced by the age of the patient or the duration of PPI therapy.

Overall, 92.2% of patients reported taking their PPI medication as prescribed, compared with a physician rating of adherence of 89.3%. This corresponded to moderate agreement between patients and physicians (κ = 0.57). There was widespread use of OTC medications for GERD. According to the physicians, 51.4% of patients with a PPI prescription also used an OTC medication for their gastrointestinal symptoms. The physicians reported that, of the 5326 patients who were recruited, 44.6% of GERD patients with a PPI prescription used antacids/alginates (8.2% often or always), 21.0% used prokinetics (3.4% often or always) and 7.1% used H_2_-receptor antagonists (0.8% often or always). There was moderate agreement between patients and physicians with respect to use of antacids or local alginates (κ = 0.44) and prokinetic agents (κ = 0.51).

**Figure 2:**
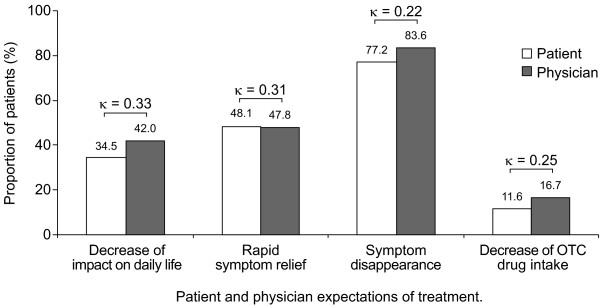
**Patient and physician ratings of patient expectations of treatment**. OTC, over-the-counter.

The majority (77.2%) of patients expected their symptoms to disappear during treatment (Figure 2), with many patients also anticipating rapid symptom relief (48.1%), improvement in daily life (34.5%) and diminished use of OTC medication (11.6%). However, there was poor agreement between patients and physicians with respect to expectations of a decreased impact on daily life, symptom disappearance, speed of symptom relief and decrease in OTC medication use during treatment, with patients tending to have lower expectations than their physicians (Figure 2).

### Residual symptoms and impact

**Figure 3:**
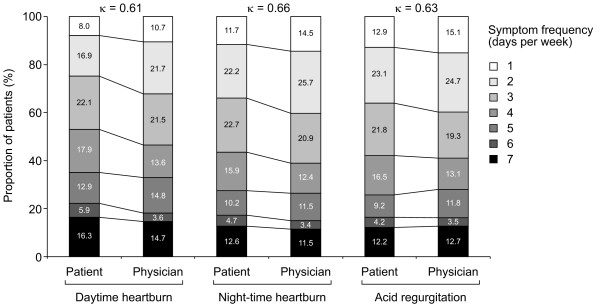
**Patient and physician ratings of the frequency of daytime heartburn, night-time heartburn and acid regurgitation in patients with each symptom**. Number of patients = 1314 for daytime heartburn, 1519 for night-time heartburn and 1302 for acid regurgitation.

Overall, 60.7% of patients reported at least one residual symptom: 41.6% reported daytime heartburn, 39.3% reported nocturnal heartburn and 36.5% reported acid regurgitation (Table 2). Physicians slightly underestimated the presence of daytime (35.8%) and nocturnal heartburn (38.8%) in patients taking PPIs, and overestimated the prevalence of regurgitation (41.3%). There was, nevertheless, good agreement between patients and physicians for both the *presence *of daytime heartburn, nocturnal heartburn and acid regurgitation during the reporting period (Table 2), and the *frequency *of daytime heartburn, nocturnal heartburn and acid regurgitation (Figure 3). It is also interesting to note the frequency of these symptoms (days per week these symptoms were experienced), as reported by patients taking PPIs. Patient-reported daytime heartburn, nocturnal heartburn and acid regurgitation occurred on five or more days per week in 26-35% of cases among those expressing these residual symptoms (Figure 3).

**Table 2:** Prevalence of gastrointestinal symptoms and patient-physician agreement.

Symptom	Presence		κ (95% CI)
	Patient	Physician	
Daytime heartburn	41.6%	35.8%	0.62 (0.59-0.64)*
Night-time heartburn	39.3%	38.8%	0.78 (0.73-0.76)†
Acid regurgitation	36.5%	41.3%	0.63 (0.61-0.65)*
Dysphagia	3.4%	4.6%	0.51 (0.45-0.567)*
Epigastric burning	15.7%	17.7%	0.65 (0.63-0.68)*
Abdominal pain	14.2%	10.6%	0.52 (0.48-0.56)*
Nausea	9.0%	7.0%	0.57 (0.53-0.61)*
Vomiting	1.4%	1.2%	0.60 (0.50-0.70)†
Bloating	26.7%	23.1%	0.67 (0.65-0.69)*
Eructation	21.7%	18.0%	0.59 (0.56-0.62)*

*P < 0.0001, McNemar's test.

†Not significant.

CI, confidence interval.

**Figure 4:**
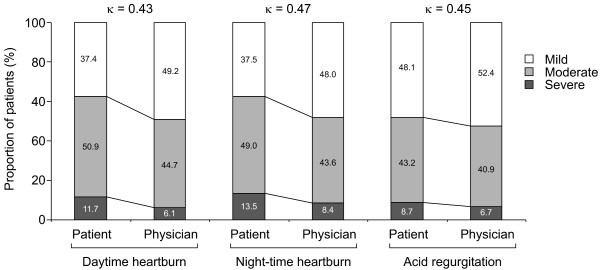
**Patient and physician ratings of the severity of daytime heartburn, night-time heartburn and acid regurgitation in patients with each symptom**. Number of patients = 1467 for daytime heartburn, 1628 for night-time heartburn and 1478 for acid regurgitation.

**Figure 5:**
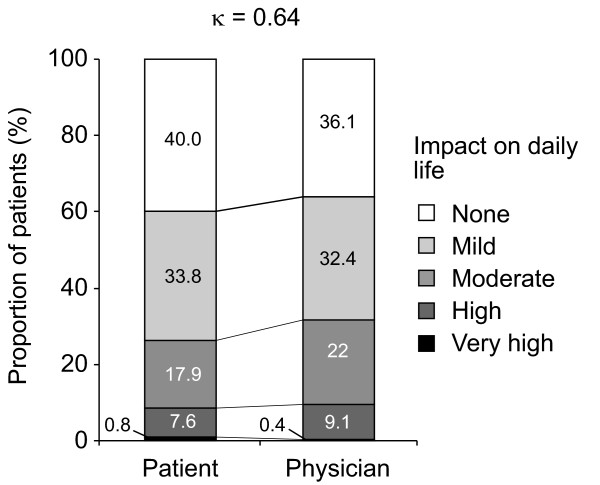
**Patient and physician ratings of the impact of reflux symptoms on daily life**.

There was only moderate agreement between patients and physicians with respect to the *severity *of residual reflux symptoms: daytime heartburn, nocturnal heartburn and acid regurgitation (Figure 4). There was good agreement between patients and physicians with respect to the effect of residual reflux symptoms on daily life (Figure 5). Residual symptoms affected the everyday lives of 60.0% of GERD patients treated with PPIs, and were rated as having a high or very high impact by 8.4% of patients (Figure 5).

## Discussion

This study shows that there is a high level of satisfaction associated with the treatment of GERD with PPIs. Patients and their physicians rated their treatment satisfaction levels as excellent or good in more than 70% of cases, and excellent, good or moderate in over 90% of cases. This compares well with previously published treatment satisfaction rates of 70-94% for GERD medical therapies [25]. Adherence to PPI prescription, as assessed by physicians and patients, was also very high at approximately 90%, although this was a simple response to a question that asked if the prescription had been followed.

Despite high satisfaction and compliance rates, just over half of all patients with GERD in the present study experienced at least one residual gastrointestinal symptom. This is consistent with previously published survey data in which 46% of daily PPI users experienced breakthrough symptoms, which occurred on 28% of treatment days [21]. Although it is difficult to determine how many patients were responders to PPI therapy in the present study it is interesting to note that many patients experiencing residual heartburn and acid regurgitation had these symptoms frequently (five or more days per week in 26-35% of cases). Furthermore, residual reflux symptoms were moderate or severe in 48-63% of cases.

There is a moderate or good degree of agreement between patients and physicians for most parameters examined in the present study. Physicians had a moderate-to-good understanding of their patients' symptom burden and satisfaction with treatment. Physicians tended to underestimate the severity of reflux symptoms by underestimating the prevalence of moderate and severe symptoms and overestimating the prevalence of mild symptoms (Figure 4). There was a poor level of agreement between patients and physicians concerning expectations of PPI treatment. In general, physicians had greater expectations than patients concerning the benefits of PPI therapy.

There are several clinical implications arising from physicians' underestimation of the severity of GERD, their high expectations of PPI therapy and the level of residual gastrointestinal symptoms observed in PPI-treated patients in this study. It may be beneficial to use patient-completed questionnaires to help improve the management of newly diagnosed and existing GERD patients in primary care [16]. Several studies show that satisfaction with treatment is associated with meeting patient expectations and good patient-physician communication [26-28]. Although satisfaction rates were high in the present study, satisfaction may be improved if physicians are able to manage patients' expectations through more effective communication. Response to therapy may be increased by educating patients about the correct way in which to take their medication to improve patient adherence [29]. A proportion of patients with GERD receiving prescription PPIs also need to move to a more effective therapy to prevent residual gastrointestinal symptoms. Additional self-medication or switching to another PPI may be helpful [30]. In addition, a therapeutic option other than acid-suppressive drugs (including surgical treatment) may prove useful, particularly in severe cases.

The data collected in the present study for treatment expectations, residual symptoms and satisfaction are similar to those of the US PUNS Study [20], the international Burning Questions survey [11] and two French treatment-satisfaction surveys conducted in primary and secondary care [18,19]. However, very few other studies have paired answers from physicians and patients within the same study. McColl and colleagues showed that before treatment, physicians tended to underestimate the severity of their patients' reflux symptoms [15]. However, after treatment, agreement between physicians and patients improved to reach a moderate to substantial level. This is similar to the findings of the present study in which all patients were receiving treatment with PPIs before the start of the study and agreement between patients and physicians was moderate. Similarly, correlations between physician and patient assessments of the severity of reflux symptoms in another study were low at baseline and stronger when assessed after treatment had begun [31].

Although the present study was a large survey of paired responses in primary care with an excellent response rate, it suffers from several disadvantages and limitations. While patients were neither specially selected nor specially invited for the purpose of this study, they may have been more likely to consult their physician if they had more severe reflux symptoms. Thus, this study may have a selection bias towards individuals with more severe symptoms. Furthermore, the high prevalence of esophagitis among patients who underwent an endoscopy in the current study could reflect a referral bias. Adherence was self-reported and so may be underestimated (particularly unintentional non-adherence such as taking the medication at the wrong time of day). Information on treatment expectations was based on patients recalling how they felt before the start of treatment. No information was available concerning patients' pre-treatment symptom burden or consultations, which may have influenced patients' satisfaction. A further possible limitation is that the study did not use a validated questionnaire, since the most appropriate tools (e.g. the Treatment Satisfaction Questionnaire [TSQ] in GERD) were not available in French. The Overall Treatment Effect (OTE) questionnaire (adapted from the Global Ratings of Change Questionnaire [GRCQ]) is validated in French, but does not capture the dimension of satisfaction [32,33].

## Conclusions

In conclusion, this study shows there were very high rates of patient-physician satisfaction and treatment adherence among GERD patients taking a PPI (all ~90%), and that there was moderate patient-physician agreement for these parameters. However, agreement levels were lower concerning therapeutic expectations, with physicians having greater expectations of PPI therapy than their patients. Residual gastrointestinal symptoms were fairly common and physicians underestimated residual symptom severity compared with their patients, though there was good agreement regarding the presence and frequency of these symptoms and their effect on patients' daily life. These results suggest the potential for better communication between physicians and their patients with GERD, possibly through structured symptom questionnaires, and a need for physicians to be open to the option of switching PPI, or choosing an alternative or additional GERD medication in selected patients with difficult-to-treat GERD.

## Competing interests

ED has participated in advisory board meetings for AstraZeneca, received fees from GSK and Sanofi Aventis for continuing medical education and from Mayoly Spindler for participation on a scientific board. J-FR has received a research grant from Olympus. CS and PB are employees of AstraZeneca. KH was an employee of AstraZeneca at the time of the study.

## Authors' contributions

All authors were involved in designing and planning the study, and in developing the manuscript. ED was a primary investigator, JF-R and KH were involved in analysing the data, and CS and PB were involved in interpretation of the data. All authors have read and approved the text as submitted.

## Pre-publication history

The pre-publication history for this paper can be accessed here:

http://www.biomedcentral.com/1471-230X/11/25/prepub
